# Emotional Intelligence, Depression, Stress and Anxiety Amongst Undergraduate Dental Students During the COVID-19 Pandemic

**DOI:** 10.3389/ijph.2023.1604383

**Published:** 2023-02-09

**Authors:** Maryati Md. Dasor, Anis Aqilah Jafridin, Aliatul Afiqah Azhar, Alhusna Abang Abdullah Asma, Prathap Chandar Manivannan, Sobia Bilal, Norashikin Yusof, Budi Aslinie Md. Sabri

**Affiliations:** ^1^ Faculty of Dentistry, Universiti Teknologi MARA, Sungai Buloh, Malaysia; ^2^ Faculty of Dentistry, Universiti Sains Islam Malaysia, Kuala Lumpur, Malaysia; ^3^ Faculty of Dentistry, MAHSA University, Jenjarum, Malaysia; ^4^ School of Dentistry, International Medical University, Kuala Lumpur, Malaysia

**Keywords:** anxiety, COVID-19, depression, stress, emotional intelligence

## Abstract

**Objectives:** This study aimed to measure depression, anxiety and stress (DAS) among undergraduate dental students during the COVID-19 pandemic, identify key contributing factors of stress and explore the association of emotional intelligence to DAS.

**Methods:** A multi-centre, cross-sectional study was conducted across four universities in Malaysia. The study administered a questionnaire consisting of the validated Depression Anxiety Stress Scale (DASS), Dental Environment Stress (DES), Emotional Intelligence Scale (EI) and 10 statements assessing COVID-19 specific potential stressor factors.

**Results:** Participants included 791 students across four universities. Abnormal levels of DAS were identified in 60.6%, 66.8% and 42.6% of the study participants, respectively. “Pressure of performance,” “Faculty administration” and “Self-efficacy belief” were the highest rated stressors. On-time graduation was the main COVID-19 specific stressor factor. EI was negatively correlated with DAS scores (*p* < 0.001).

**Conclusion:** The levels of DAS during COVID-19 pandemic in this population is high. However, participants with higher EI presented with lower DAS suggesting that EI may be a form of coping resource and should be enhanced in this population.

## Introduction

The pandemic of novel coronavirus disease 2019 (COVID‐19) is perhaps the worst public health epidemic of the 21st century [1]. Numerous attempts to mitigate disease spread have been carried out on a global scale, leaving no aspect of daily life unaffected. Across the globe, many countries attempted to contain the spread of COVID-19 by implementing some form of cordon sanitaire, or a Movement Control Order (MCO). To this end, the Malaysian government implemented the first of a series of MCOs on the 18th of March 2020 [2]. The MCO involves the prohibition of mass movements and gatherings at all places nationwide. It includes the closure of all business premises with the exception of essential services [3, 4]. Despite many institutions ceasing physical classes and switching to online learning, closing campuses and sending students home, students were not exempt from this disruption [5]. Moreover, a slew of other events, including conferences, exhibitions, sporting events and graduations were cancelled and moved to online avenues [6]. Furthermore, and more importantly, the pandemic resulted in a disruption of clinical training leaving students with incomplete clinical requirements which may affect graduation [7]. It is imperative to appreciate the unique challenges these trying times present for the educational experience of students and the impact this challenge may have on them. Previous research on the psychological effects of outbreaks of communicable diseases such as MERS and SARS on students discovered an increase in the incidence of mild to moderate anxiety. However, minimal research has been carried out since the COVID-19 outbreak to assess the pandemics’ influence on students’ psychological wellbeing and none have been conducted on undergraduate dental students in Malaysia. Depression, stress, and anxiety are all important indicators of psychological health. Depression, stress, and anxiety affect approximately 2.3 million Malaysians [8] with moderate to severe levels of DAS ranging from 13.9% to 29.3%, 51.5%–55.0%, and 12.9%–21.6% among undergraduates, respectively [9].

Among health professions, dental students have been reported to be more prone to psychological problems (stress, anxiety and depression) and high mental burden even prior COVID-19 pandemic due to the technical nature of the work requiring thorough knowledge of biological sciences in relation to clinical work [10–12].

Studies have found that dental students have higher levels of stress as compared to the general population [13, 14]. Dentistry has been classified among the toughest, most challenging, and exhausting courses of to study, with dental students required to possess a wide range of competencies such as academic and clinical capabilities, including social skills [15]. The dental curriculum in Malaysia consists of a 2-year preclinical didactic and laboratory course followed by 3 years of clinical training [16]. The learning outcomes of the undergraduate dental education should reflect the full range of knowledge, skills, attitudes and upon graduation, the dental graduate’s need to be ready to contribute to the general health of the population by being capable of providing basic dental treatment and implement and promote appropriate oral health management to his/her patients and communities in a culturally sensitive manner’ (MPM137) [17]. In order to achieve this goal, dental undergraduates need to undergo a rather challenging pedagogical experience.

Dental undergraduate training includes performing operative procedures on patients under the supervision of experienced clinicians and academics. Students also have a moral responsibility to their clinical work and to develop professionalism, ethics and team-working skills in the course of their undergraduate training (Ali et al 2017) [18].

According to studies, students who were subjected to high levels of stress on a consistent basis were emotionally exhausted, as well as experiencing mental distress which can manifest as physical symptoms and, ultimately result in burnout [19].

Female gender, year of study, student’s age and staying in the hostel are positive predictors for severity of stress [20]. The dental students who experiencing higher psychological health complaints were predominantly female (nervousness, sleeplessness, anxiety, depression and mental exhaustion) [12, 21]. Contrastingly, a systematic review found that male students have higher odds for burnout which is a result of high-level, prolonged stress rather than other psychological problems [22].

The trend of psychological problems incrementally increases throughout their education with clinical students and interns having higher prevalence rate of depression, anxiety and stress compared to preclinical students [20, 23].

In addition, the students from government colleges showed higher prevalence of DAS than their counterparts from private colleges. A similar finding was observed among students staying in the hostel compared to those living with their family [20].

Students bring with them to school certain background characteristics such as their racial/ethnic and linguistic identities and, sometimes, personal or family problems that affect their ability to learn. Private universities are known to have students who come from higher socioeconomic status (SES) with families’ annual incomes typically over $50,000 USD compared to public school students whose household income can be less than $15,000 USD [24].

While the credibility of studies looking at the relationship between suicide and occupation showing high suicide rates amongst dentists as compared to other professions and to the general public has been challenged, stress and depression among healthcare professionals are still major concerns as both have been shown to affect the quality of treatment provided and impacting the safety of the patient [25, 26]. A Swedish study on perceived stress in adults highlighted that clinicians need to be aware of the suicidal risk associated with high levels of stress [27]. Hence, the signs of stress among dental students must not be ignored.

Meanwhile, research also showed that dental undergraduates who have high emotional intelligence (EI) are less likely to report perceived stress [28]. A multi-institutional and multi-profesional survey showed similar findings indicating EI helps with moderating stress at lower levels [29]. Emotional perception, assessment, and expression; emotional ease of thought; understanding, analysing, and applying emotional knowledge; and the ability to reflectively regulate one’s own emotions defined EI [30]. In layman’s terms, EI can be understood as the extent of an individual’s capability to interpret and control emotions in themselves, and other people, and manage relations more effectively. Thus, highly emotionally intelligent students are predicted to be able to cope with learning environment stressors better and to experience lower stress [28] EI has been suggested as a key component of professional skills in the medical curriculum. The role of EI in enhancing patient outcomes has been recognized which has led to it being included as a part of the undergraduate dental student preadmission criteria.

However, little research has investigated the role of EI in the relationship between stressors and DAS levels in dental undergraduates, particularly in Malaysia. Hence the objectives of this research study is to estimate the prevalence rates and levels of DAS among undergraduate dental students during COVID-19 pandemic, identify key contributing factors to stress and explore the association between emotional intelligence and depression, stress, and anxiety among undergraduate dental students. The findings of this research can be used to create an academic environment which takes into consideration sources of stressors that are significant in this population in high stress environment like a pandemic produces.

## Methods

### Design

A cross-sectional survey was conducted among Malaysian undergraduate dental students from four major universities: Universiti Teknologi MARA (UiTM) (*n* = 416), Universiti Kebangsaan Malaysia (UKM) (*n* = 237), MAHSA University (MAHSA) (*n* = 366), and International Medical University (IMU) (*n* = 295) from 26 November 2020 to 31 December 2020 which is during the early phase of the pandemic.

These four universities were chosen based on a purposeful sampling method. Selection was based on geographical location, whereby all the universities were in the Klang Valley and subjected to similar restrictions during the pandemic. These four universities also had the highest number of students, consequently providing the most respondents. The number of the private and public universities are equally selected to ensure a normal distribution of the sample in terms of ethnicity and socioeconomic status.

In Malaysia, the undergraduate dental curriculum is governed by the professional bodies namely; Malaysian Dental Council and the Malaysian Qualifications Agency which determines the programme standard and structure across the four universities. Therefore, the admitting academic qualification (level of IQ) and the programme requirements are similar throughout the dental schools.

This study was conducted in accordance with the 1964 Helsinki declaration and its later amendments. Ethics approval and permission to conduct the study were obtained from UiTM [REC/04/2020 (MR/79)], UKM (PPI/111/8/JEP-2020-402), MAHSA (RMC/EC39/2020) and IMU (4.18/jcm-212/2020), respectively. This study involved all undergraduate dental students including extended year students. Extended year students are year 5 students who continued for another 6 months clinical training to complete their cases before they graduated. This study applied a census sampling method, however the minimum sample size calculated to achieve a 95% confidence interval with a 5% margin of error and a 30% drop out rate was 390. A total of 719 undergraduate dental students completed the online questionnaire. The response rate ranged from 40% to 85% across the 4 universities. Of these, 284 (39.4%) were from Universiti Teknologi MARA, 135 (18.7%) were from Universiti Kebangsaan Malaysia, 177 (24.6%) were from MAHSA University and 124 (17.2%) were from International Medical University. This study did not manage to collect information on reasons for non-participation.

### Measures

Data was collected *via* a self-reported online questionnaire that included an initial section with information about the research and any associated risks, a brief explanation on how to answer the questionnaire and an online consent form for the students to fill in before proceeding with the questionnaire. The questionnaire also specified that participation was entirely voluntary.

The questionnaire was made up of five sections. The first section (Part A) included demographic questions regarding age, gender, name of program, year of study, marital status, study status, the decision of dentistry as a first choice, scholarship support and household income. The second section (Part B) comprised of the Depression Anxiety Stress Scale (DASS-21). The students were required to rate items on a scale of 0 (did not apply to me at all) to 3 (applied to me very much or most of the time). The third section (Part C) assessed contributing factors to stress among students using Dental Environment Stress (DES) Questionnaires. Students are required to choose and rate potential stressors, which are listed and include factors related to academic, personal, and clinical/laboratory environments. The fourth section (Part D) contained COVID-19 Pandemic Related Concerns which is comprised by 10 statements that are concerned with the potential stress factors that dental students may face during the COVID-19 pandemic. Stressors have been documented to be strong risk factors for several mental health issues including depression and anxiety [31, 32]. The fifth section (Part E) contained the Schutte Self Report Emotional Intelligence Test (SSEIT) which assesses emotional intelligence using thirty-three items, three of which are reverse-scored and measured on a five-point Likert scale from 1 to 5. The possible scores ranged from 33 to 165, with 33 indicating low EI and 165 indicating high EI [33].

The DASS-21 is an updated version of the original forty-two items survey (DASS-42). It is made up of three self-reported scales intended to assess an individual’s negative emotional states of DAS. It has been translated into various languages and validated in different populations including Malaysia. This tool contains twenty-one items, seven items for each emotional state, divided into subscales with similar contents. Dysmorphia, hopelessness, involvement, anhedonia, and inertia are all assessed using the Depression scales. The Anxiety scale measures psychological arousal, skeletal muscle effects, perceived stress, and subjective experience of anxious effect. The Stress scale measures difficulty in relaxing, anxious arousal, susceptibility to agitation, irritability over-reactive state, and frustration.

The Dental Environment Stress (DES) questionnaire has been widely applied in many studies with comparable goals previously as it is a validated questionnaire [14, 23–27]. The DES questionnaire consists of 38 questions pertaining to potential stressors across five parts (A-E) [27]. The 38 questions are grouped into seven sub-categories in the DES such as personal issues (PI), academic performance (AP), educational environment (EE) and learning clinical skills (LCS). The items are measured on a Likert scale of seven points with 1 = not stressful at all, and 7 = very stressful.

Part D of the study instrument consisted of 10 questions which focused on the students’ wellbeing in adapting to teaching and learning online during the early phase of the pandemic and their concerns with contracting the disease when they are face-to-face with various people. They are based on Hung’s validated questionnaire and modified to suit the scenario of the local undergraduates [32]. A four-point Likert scale from 1 to 4 was used to measure the level of stress in facing such situations, with a score of 1 indicating “not stressful at all” and a score of 4 indicating “very stressful.” Due to the pandemic, the pilot study was conducted online with 15 students: 8 students from Faculty of Dentistry, UiTM and 7 students from Faculty of Dentistry, UKM. The items in this questionnaire are tabulated in Table 4.

Emotional intelligence (EI) was measured using a scale developed by Schutte et al. [33] which was also tested for its’ structure, predictive and discriminative validity [31]. The EI scale has thirty-three items, three of which are graded in reverse, calculated on a five-point Likert scale from 1 to 5. The possible range of scores is 33, indicating low EI, to 165, indicating high EI.

The online questionnaire link was shared with all undergraduate class representatives from each university and distributed to participants. The questionnaire was administered during the movement control order which took place in the last quarter of 2020 and affected all the universities in this study.

### Analysis

Data analysis was performed using Statistical Package for Social Sciences (SPSS) version 25 software. Descriptive statistics were reported based on the types and distribution of the data. Categorical data were shown as frequencies and percentages, while numerical data were shown as means and standard deviations. Two-way analysis of variance (ANOVA) was used to analyse the responses, and statistical significance was set at less than 0.05. Logistic regression analysis was performed to test the association of EI, socio-demographic characteristics such as age, gender, institution of study, year of study and current accommodation status with depression, anxiety and stress.

## Results

A total of 719 undergraduate dental students completed the online questionnaire. The response rate ranged from 40% to 85% across the 4 universities. Of these, 284 (39.4%) were from Universiti Teknologi MARA, 135 (18.7%) were from Universiti Kebangsaan Malaysia, 177 (24.6%) were from MAHSA University and 124 (17.2%) were from International Medical University. Table 1 shows the demographic of participants result summary. The average age of respondents was 21 years old which ranges from 17 to 28 years old. Majority of respondents were female (81.0%), single (99.7%) and were clinical year students (57.9%). A majority of the sample were also living at home (71.1%) during the period of the study and had indicated that dentistry was their first choice of degree program (90.7%).

**TABLE 1:** Socio-demographic distribution of respondents. (Emotional Intelligence, Depression, Stress and Anxiety Amongst Undergraduate Dental Students during COVID-19 Pandemic (Malaysia, 2019–2021).

Sample characteristics	Unweighted (N = 719)	
	n	%
Age (Mean ± SD)	21.66 ± 2.011	
Gender		
Female	582	80.9
Male	137	19.1
Marital status		
Single	717	99.7
Married	1	0.1
Divorce	1	0.1
Year of study		
Year 1	151	21.0
Year 2	152	21.1
Year 3	132	18.4
Year 4	152	21.1
Year 5	128	17.8
Extended year 5	4	0.6
Study status		
Normal	680	94.6
Extended (i.e.: Year 3 extended due to repeat year or barred due to requirement)	39	5.4
Accommodation status		
At home	511	71.1
University rented	101	14.0
Private rented	107	14.9
Dentistry as a 1st choice		
Yes	652	90.7
No	67	9.3

Table 2 shows the prevalence of DAS by severity (five-categories). In terms of depression, 39.4% of the respondents were rated as normal while the majority of the respondents reported some form of depression. Similarly, in terms of anxiety, 33.2% of the respondents were reported as normal while the majority reported some form of anxiety. In terms of stress, the majority of respondents (57.4%) reported normal stress levels. No significant association was found between depression, anxiety, and stress and study status, marital status, financial support, household income, accommodation situation and if dentistry was a first choice (*p* > 0.05). A significant association was found between gender and anxiety where females reported higher mean anxiety scores (12.41 SD ± 8.82) as compared to the males (9.72 SD ± 7.32) (*p* = 0.07). A significant association was also found between year of study and anxiety where extended year 5 students reported higher anxiety scores as compared to other clinical years (*p* < 0.05).

**TABLE 2:** Severity ratings of depression, anxiety, and stress (Emotional Intelligence, Depression, Stress and Anxiety Amongst Undergraduate Dental Students during COVID-19 Pandemic (Malaysia, 2019–2021).

Severity	Depression		Anxiety		Stress	
	n	%	n	%	n	%
Normal	283	39.4	239	33.2	413	57.4
Mild	125	17.4	78	10.8	108	15.0
Moderate	187	26.0	178	24.8	105	14.6
Severe	55	7.6	92	12.8	67	9.3
Extremely severe	69	9.6	132	18.4	26	3.6

The DES questionnaire was analysed by item and according to the seven domains (Supplementary Table S1). The six items with the highest mean scores are indicated with an asterisk. Item “Fear of failing course or year” yielded the highest score (3.38 SD ± 0.89) while “self-efficacy beliefs” (2.65 SD ± 0.86) and “performance pressure” (2.99 SD ± 0.89) were the two domains which scored highest on the DES-scale among the average. The domains with the lowest mean score were “other/personal factors” (1.60 SD ± 0.86) and “faculty and administration” (2.04 SD ± 0.86).

With regards to the COVID-19 related factors (Supplementary Table S2), the main stressor was concerning on-time graduation (3.09 SD ± 0.95) followed by lack of focus and motivation during online learning (2.77 SD ± 0.99) and emotional health (2.65 SD ± 0.9).

The relationship between emotional intelligence and DAS among undergraduate dental students shows a moderate negative correlation between EI and depression (*p* < 0.001) while a weak negative correlation was found between EI and stress and anxiety score (*p* < 0.001) (Supplementary Table S3). Depression was moderately positively correlated with anxiety and stress (*p* = 0.001), while anxiety and stress were found to have a strong positive correlation (*p* = 0.001).

Logistic regression was performed to ascertain the association between emotional intelligence score, institution of study, current residence, year of study and on the likelihood of reporting depression, stress, and anxiety. For this analysis, the depression, stress, and anxiety scores were dichotomised to normal cases and cases reporting mild to severe depression, stress, and anxiety, respectively. The logistic regression for all three models were statistically significant, Hosmer-Lemeshow test; (Chi-square 5.142 df = 8, *p*-value = 0.742), (Chi-square 13.794 df = 8, *p*-value = 0.087), (Chi-square 8.960 df = 8, *p*-value = 0.346) respectively indicated a good model fit.

The model explained 12.8% (Nagelkerke R2) of the variance in depression and correctly classified 63.4% of cases. Meanwhile, the model for anxiety explained 5.6% (Nagelkerke R2) of the variance in anxiety and correctly classified 66.2% of cases. The model for stress explained that 6.4% (Nagelkerke R2) of the variance in stress and correctly classified 62.4% of cases. Higher emotional intelligence score indicated a lower probability of reporting mild to severe depression, stress, and anxiety in all three models (Table 3).

**TABLE 3:** Binary Logistic Regression Analyses: predicting depression, stress and anxiety (Emotional Intelligence, Depression, Stress and Anxiety Amongst Undergraduate Dental Students during COVID-19 Pandemic (Malaysia, 2019–2021).

	B	S.E	Wald	df	Sig	Exp (B)	95% confidence interval	
							Lower	Higher
Depression								
E.I Score	−0.050	0.007	52.94	1	0.000	0.951	0.938	0.964
Stress								
E.I Score	−0.031	0.006	25.816	1	0.000	0.969	0.958	0.981
Anxiety								
E.I Score	−0.023	0.006	13.426	1	0.000	0.977	0.965	0.989

Socio-demographic characteristics such as age, gender, institution of study, year of study and current accommodation status did not contribute significantly to all three models.

Tables 4–6 show that in the fully adjusted model, emotional intelligence is significantly positively associated with anxiety, depression, and stress, (OR0.51; 95%CI: 0.27–0.97). It was apparent from the unadjusted model 1 in Tables 4–6 that having low or moderate emotional intelligence had a risk effect on anxiety, depression and stress characterized as having abnormal DAS scores.

**TABLE 4:** Hierarchical logistic regression models for the associations of emotional intelligence, gender, household income and financial support with anxiety characterized as having abnormal DAS score for anxiety (Emotional Intelligence, Depression, Stress and Anxiety Amongst Undergraduate Dental Students during COVID-19 Pandemic (Malaysia, 2019–2021).

Variables	Model 1	Model 2	Model 3	Model 4
Emotional Intelligence				
High	1	1	1	1
Low	2.16 (1.15–4.07)*	2.21 (1.17–4.16)*	2.45 (1.29–4.65)*	2.43 (1.28–4.63)*
Moderate	1.04 (0.60–1.80)	1.06 (0.61–1.83)	1.11 (0.64–1.94)	1.12 (0.64–2.00)
Gender				
Male		1	1	1
Female		1.37 (0.93–2.02)	1.26 (0.85–1.88)	1.29 (0.86–1.92)
Household income				
>RM13,148			1	1
≤RM 3000			0.74 (0.45–1.21)	0.76 (0.46–1.27)
RM3001–RM6274			0.72 (0.44–1.18)	0.73 (0.44–1.21)
RM6275–RM13,148			1.29 (0.79–2.11)	1.31 (0.81–2.14)
Financial support				
Self-funding				1
Scholarship				0.79 (0.55–1.15)
Loan				0.95 (0.61–1.46)

*p ≤ 0.05, **p ≤ 0.001.

Model 1: Emotional Inteligence.

Model 2: Variables in model 1 plus gender.

Model 3: Variables in model 2 plus household income.

Model 4: Variables in model 3 plus financial support.

**TABLE 5:** Hierarchical logistic regression models for the associations of emotional intelligence, gender, household income and financial support with depression characterized as having abnormal DAS score for depression (Emotional Intelligence, Depression, Stress and Anxiety Amongst Undergraduate Dental Students during COVID-19 Pandemic (Malaysia, 2019–2021).

Variables	Model 1	Model 2	Model 3	Model 4
Emotional Intelligence				
High	1	1	1	1
Low	7.06 (3.72–13.41)**	7.23 (3.80–13.77)**	7.29 (3.82–13.94)**	7.29 (3.80–13.99)**
Moderate	2.12 (1.22–3.67)*	2.16 (1.25–3.73)*	2.16 (1.25–3.75)*	2.21 (1.27–3.85)*
Gender				
Male		1	1	1
Female		1.38 (0.93–2.04)	1.39 (0.94–2.07)	1.45 (0.97–2.16)
Household income				
>RM13,148			1	1
≤RM 3000			0.89 (0.45–1.45)	0.93 (0.56–1.54)
RM3001–RM6274			1.07 (0.65–1.75)	1.08 (0.66–1.80)
RM6275–RM13,148			0.99 (0.63–1.59)	1.01 (0.63–1.61)
Financial support				
Self funding				1
Scholarship				0.63 (0.44–0.91)*
Loan				1.07 (0.70–1.66)

*p ≤ 0.05, **p ≤ 0.001.

Model 1: Emotional Inteligence.

Model 2: Variables in model 1 plus gender.

Model 3: Variables in model 2 plus household income.

Model 4: Variables in model 3 plus financial support.

**TABLE 6:** Hierarchical logistic regression models for the associations of emotional intelligence, gender, household income and financial support with stress characterized as having abnormal DAS score for stress (Emotional Intelligence, Depression, Stress and Anxiety Amongst Undergraduate Dental Students during COVID-19 Pandemic (Malaysia, 2019–2021).

Variables	Model 1	Model 2	Model 3	Model 4
Emotional Intelligence				
High	1	1	1	1
Low	3.13 (1.69–5.82)**	3.21 (1.72–5.99)**	3.34 (1.78–6.26)**	3.32 (1.77–6.22)**
Moderate	1.37 (0.77–2.42)	1.39 (0.79–2.47)	1.43 (0.80–2.54)	1.44 (0.81–2.57)
Gender				
Male		1	1	1
Female		1.47 (0.99–2.18)	1.40 (0.94–2.08)	1.42 (0.95–2.12)
Household income				
>RM13,148			1	1
≤RM 3000			0.96 (0.59–1.55)	0.99 (0.61–1.61)
RM3001–RM6274			0.74 (0.45–1.20)	0.75 (0.46–1.22)
RM6275–RM13,148			1.03 (0.65–1.62)	1.04 (0.66–1.63)
Financial support				
Self funding				1
Scholarship				0.79 (0.55–1.12)
Loan				0.99 (0.66–1.50)

*p ≤ 0.05, **p ≤ 0.001.

Model 1: Emotional Inteligence.

Model 2: Variables in model 1 plus gender.

Model 3: Variables in model 2 plus household income.

Model 4: Variables in model 3 plus financial support.

The probability ratio of experiencing abnormal DAS scores was OR 2.16; 95% CI: 1.15–4.07 (anxiety), OR 7.06; 95% CI: 3.72–13.41(depression) and OR 3.13; 95% CI:1.69-5.82 (stress), respectively in the low EI group as compared to the high EI group. The ratio of probability increased after adjusting for gender, household income and financial support for all three DAS outcomes.

## Discussion

The demographic distribution of the sample reflects the actual demographic distribution of dental students enrolled in the four universities, enhancing the generalizability of the findings of this study. Among the 719 respondents, the prevalence of DAS was 60.6% (depression) 66.8% (anxiety) and 42.6% (stress), respectively. This prevalence of abnormal levels of DAS and severity is higher in this population as compared to a previous study utilising similar instruments among other university students in Malaysia [34] but were similar to students in Saudi Arabia [14]. The prevalence is high because, in addition to dealing with usual day-to-day stressors of being a student, dental students must deal with stressors specific to dental school, including adapting to the clinical training, completion of the number of patients in combination and gaining competence in academic [35]. The higher prevalence and severity can also most likely be attributed to the additional stressor posed by the pandemic which discontinuation of traditional methods of education, substituted with written online assignments, webinars, and computer-based examinations. Additional findings also can be explained by the e-learning dental education, the lack of social interaction among peers due to numerous movement control orders and social distancing, and the absence of clinical sessions to complete clinical requirements [36].

In this study female students reported higher levels of stress and anxiety, and while this is consistent with findings from other studies [13, 37, 38], the existing literature explaining the gender gap in anxiety is not conclusive. The gender gap can possibly be explained by the methodological artifact explanation which suggests that females tend to be socialized to verbalize their stresses and feelings more openly than men; at the same time, socio-cognitive explanations of gender differences in health and stress show that gender roles and traits (masculinity in particular) explain part of the gender disparity in stress, notably cognitive appraisal whereby traditional socialization is advantageous for men in terms of health [39, 40]. The vulnerability argument suggests that females may lack coping resources, such as high self-esteem, a sense of coherence, or appropriate coping strategies for handling the stressors [40].

While other studies reported that those whose first choice of programme was dentistry reported less stress, there was no significant association noted within this study [13, 37, 38].

In the current study, marital status and financial responsibilities were not found to be significant predictors of DAS. Similarly, other studies [38, 41] reported that marital status is not a significant predictor of academic success. While being married may be a source of stress due to the expanded duties, it may at the same time be a source of support or coping mechanism through the protective effect which marital relationship and care of children may also yield. However, outcomes must be interpreted with caution as the percentage of married respondents in this study was very small. The high levels of DAS may well be credited to the burden applied on undergraduates through their workload, clinical requirements, examinations, and grade related stress [38, 42]. Findings in this study indicate that stress was essentially academic-related. Similar findings were reported by other studies [43, 44]. In this study, factors related to self-efficacy belief and faculty and administration been observed to be the main stressor undergraduate dental students. The most stressful factor for dental students was identified as “fear of failing a course or year,” followed by “completing graduation requirements,” “fear of being unable to catch up if behind,” and “lack of confidence to be a successful dentist.” A possible explanation for this high prevalence is the number and intensity of daily stressor dental students face such as the expectation to be prepared to manage a diverse complement of patients. Students may experience stress due to their lack of preparation and their clinical lecturers’ high expectations. Students who lacked confidence may spend more time thinking and worrying about what other classmates have achieved than focusing on their own competence and potential. Subsequently, those who have poor time management skills may feel the burden of clinical requirement and academic exams as a major source of stressor. College students are among those considered most vulnerable to mental health concerns.

Our findings propose a significant negative association between the COVID-19 pandemic and a variety of academic, physical, health and emotional-related results. The findings suggest that the majority of the students experienced increased stress and anxiety due to the COVID-19 pandemic. The foremost prominent among students was concerning graduating on time. This can most likely be attributed to the issues regarding completing graduation requirements, especially preclinical and clinical requirements due to the limitations imposed by the MCO. Although the majority of students were concerned about their academic performance, nearly half of the participants were also concerned about their emotional health due to limited physical interactions with their friends and family members. During this pandemic, dental students might face difficulties with in-person interactions such as in-person meetings as well as a restriction on doing outdoor activities (e.g., jogging, hiking) to express their feelings and try to cope with stress.

This study also found that emotional intelligence was negatively correlated with depression, stress, and anxiety, consistent with previous studies [45]. After controlling for potential confounders such as age, gender and year of study, a higher emotional intelligence score was still found to account for a lower probability of reporting mild to severe depression, stress, and anxiety. This finding suggests that emotional intelligence is a coping resource that may serve as a protective factor for stress, depression and anxiety which is consistent with previous studies [21]. Despite the ongoing debate about whether EI is modifiable, there is some evidence supporting its potential for enhancement [39]. Hence it may be valuable to investigate further potential strategies for addressing the stress in dental students, such as the application of EI assessment during student selection and interventions to enhance EI.

The findings of this study should be interpreted taking into consideration the limitations of the study. The result of this study may not represent the entire Malaysian student body as the study only included 4 out of the 13 dental schools in Malaysia. The names of which were omitted to comply with confidentiality for the universities involved in this study. This is to ensure no negative perception towards the reaction of the universities during the pandemic especially pertaining to student support. Data collection was also done during the second movement control order in Malaysia during the last quarter of 2020, however the COVID-19 situation in Malaysia peaked in the months following data collection. Levels of DAS and the concerns may have differed had the data collection been done later. The difference between the response rates could possibly be attributed to the fact that data collection was led by co-researchers who were also faculty members for the three universities with the lower response rates and in contrast, the data collection at the university with the highest response rate was carried out by co-researchers who were students which may have encouraged the participants who were also fellow students to participate in the study as a show of support. Furthermore, considering that DAS can still be considered a sensitive issue in the Malaysian culture, there may be uncertainty in terms of the accuracy of the reporting by respondents. In addition, due to the anonymity employed in this study, it was not feasible to compare the socioeconomic distribution of non-responders to respondents or to ascertain the reasons of not participating among non-responders, adding to the possibility of bias in the findings.

### Conclusion

In conclusion, during the COVID-19 pandemic, dental undergraduates experienced high levels of DAS which can be attributed to factors such as performance pressures, faculty and administration, self-efficacy, and on-time graduation. Participants with higher emotional intelligence, on the other hand, reported lower levels of DAS, implying that emotional intelligence may serve as a type of coping resource. This finding emphasises the importance of providing support programmes and implementing preventive measures, particularly those that foster emotional intelligence in order to help those who have predispositions for depression, anxiety, and stress. Further research should be conducted to explore the exact role emotional intelligence plays in the pathway between stressors and depression, stress, and anxiety experience.
