# Supplementary material for: Emotional Intelligence, Depression, Stress and Anxiety Amongst Undergraduate Dental Students During the COVID-19 Pandemic
Source: Int J Public Health. 2023 Feb 9;68:1604383. doi: 10.3389/ijph.2023.1604383 (PMC9947836; doi:10.3389/ijph.2023.1604383)
Supplement: Supplementary file 1 [file Table1.DOCX]

**Supplementary File 1** | Mean scores for Dental Environment Stress Items. (Emotional Intelligence, Depression, Stress and Anxiety Amongst Undergraduate Dental Students during COVID-19 Pandemic. (Malaysia,2019-2021)

| **DES Items** | **Mean** | **SD** |
| --- | --- | --- |
| **Self-efficacy beliefs** | **2.65** | **0.855** |
| *Lack of confident to be a successful dental student* | 2.83 | 0.804 |
| *Lack of confident to be a successful dentist** | 2.89 | 0.812 |
| *Insecurity concerning professional future* | 2.81 | 0.864 |
| *Considering entering some other fields of work* | 2.07 | 0.938 |
| **Faculty and administration** | **2.04** | **0.865** |
| *Atmosphere created by clinical faculty* | 2.54 | 0.924 |
| *Amount of cheating in dental school* | 1.71 | 0.805 |
| *Rules and regulation in dental school* | 2.22 | 0.964 |
| *Expectation of dental school and what is it like** | 2.84 | 0.926 |
| *Lack of input into the decision-making process of dental school* | 2.32 | 0.917 |
| *Attitude of school towards female dental students* | 1.49 | 0.770 |
| *Relations with members of the opposite sex* | 1.44 | 0.709 |
| *Discrimination due to race, class status or ethnic group* | 1.41 | 0.722 |
| *Inconsistency of feedback on work between different instructors* | 2.36 | 0.967 |
| **Workload** | **2.61** | **0.916** |
| *Amount of assigned classwork* | 2.49 | 0.897 |
| *Lack of time for relaxation* | 2.72 | 0.934 |
| **Patient treatment** | **2.20** | **0.936** |
| *Lack of cooperation by patient in their home care* | 2.13 | 0.911 |
| *Responsibilities for comprehensive patient care* | 2.22 | 0.936 |
| *Patient being late or not showing for their appointment* | 2.58 | 1.056 |
| *Working on patients with dirty mouths* | 1.88 | 0.842 |
| **Clinical training** | **2.62** | **0.906** |
| *Difficulty in learning clinical procedures* | 2.57 | 0.897 |
| *Difficulty in learning precision manual skills required in preclinical and laboratory work* | 2.67 | 0.915 |
| **Performance pressure** | **2.99** | **0.888** |
| *Difficulty of classwork* | 2.41 | 0.846 |
| *Competition for grades* | 2.75 | 0.976 |
| *Examination and grades** | 3.15 | 0.870 |
| *Fear of failing course or year ** | 3.38 | 0.892 |
| *Complete graduation requirements** | 3.31 | 0.852 |
| *Fear of being unable to catch up if behind ** | 3.29 | 0.865 |
| *Receiving criticism about work* | 2.62 | 0.914 |
| **Other/personal factors** | **1.60** | **0.860** |
| *Having children at home* | 1.47 | 0.837 |
| *Marital adjustment problems* | 1.40 | 0.780 |
| *Financial responsibilities* | 2.12 | 1.018 |
| *Forced postponement of marriage or engagement* | 1.37 | 0.761 |
| *Lack of confident in career decision* | 2.39 | 0.968 |
| *Personal physical health* | 2.08 | 0.927 |
| *Necessity to postpone having children* | 1.30 | 0.646 |
| *Lack of home atmosphere in living quarters* | 1.74 | 0.925 |
| *Having a dual role of spouse/parent and dental student* | 1.50 | 0.881 |
