# Supplementary material for: Emotional Intelligence, Depression, Stress and Anxiety Amongst Undergraduate Dental Students During the COVID-19 Pandemic
Source: Int J Public Health. 2023 Feb 9;68:1604383. doi: 10.3389/ijph.2023.1604383 (PMC9947836; doi:10.3389/ijph.2023.1604383)
Supplement: Supplementary file 2 [file Table2.DOCX]

**Supplementary File 2** | Mean scores for COVID-19 related stressor items. (Emotional Intelligence, Depression, Stress and Anxiety Amongst Undergraduate Dental Students during COVID-19 Pandemic. (Malaysia,2019-2021)

| **Items** | **Mean** | **Standard Deviation** |
| --- | --- | --- |
| Comfort adapting to online learning | 2.37 | 0.95 |
| Quality of online teaching | 2.28 | 0.90 |
| Focus and motivation to online learning | 2.77 | 0.99 |
| Completion of your degree program on time | 3.09 | 0.94 |
| Your physical health | 2.14 | 0.95 |
| Your emotional health | 2.65 | 0.99 |
| Contracting COVID-19 while treating patients | 2.60 | 1.04 |
| Contracting COVID-19 while attending classes on campus | 2.63 | 1.04 |
| Contracting COVID-19 while interacting with people in campus | 2.61 | 1.02 |
| Financial stability | 2.16 | 0.99 |
