# Supplementary material for: Emotional Intelligence, Depression, Stress and Anxiety Amongst Undergraduate Dental Students During the COVID-19 Pandemic
Source: Int J Public Health. 2023 Feb 9;68:1604383. doi: 10.3389/ijph.2023.1604383 (PMC9947836; doi:10.3389/ijph.2023.1604383)
Supplement: Supplementary file 3 [file Table3.DOCX]

**Supplementary File 3** | Correlation matrix of Emotional Intelligence, Depression, Stress and Anxiety. (Emotional Intelligence, Depression, Stress and Anxiety Amongst Undergraduate Dental Students during COVID-19 Pandemic. (Malaysia,2019-2021)

|  | **1** | **2** | **3** | **4** |
| --- | --- | --- | --- | --- |
| Emotional intelligence | 1 |  |  |  |
| Depression | -0.318** |  |  |  |
| Anxiety | -0.158** | 0.665** |  |  |
| Stress | -0.238** | 0.775** | 0.806** | 1 |

** *Correlation is significant at the 0.01 level (2tailed)*
